# Supplementary material for: Role of DNA modifications in Mycoplasma gallisepticum
Source: PLoS One. 2022 Nov 22;17(11):e0277819. doi: 10.1371/journal.pone.0277819 (PMC9681074; doi:10.1371/journal.pone.0277819)
Supplement: S1 Fig — RM system MgaS6I is marked in orange, variable lipoproteins vlhA clusters in green, the CRISPR system in blue. M. gallisepticum S6 genome is on the top, below the genomes of the other strains. A–whole genome representation, B–fragment of genomes near MgaS6I genomic context. (PDF) [file pone.0277819.s001.pdf]

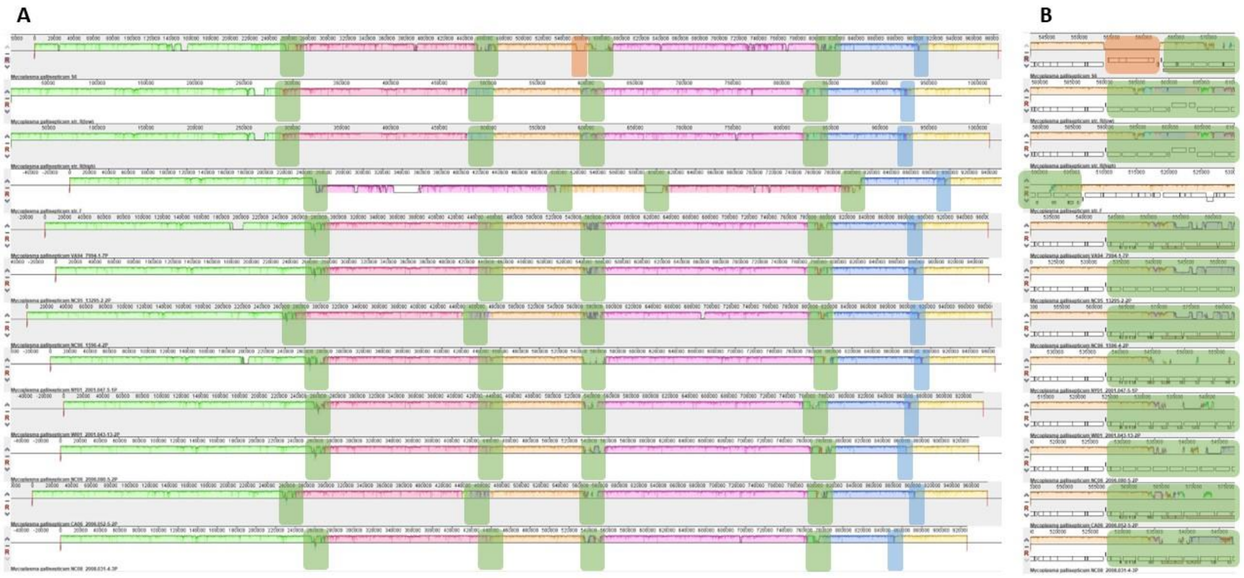

Figure S1. **Genome alignment of Mycoplasma gallisepticum strains obtained using the program Mauve.** RM system MgaS6I is marked in orange, variable lipoproteins vlhA clusters in green, the CRISPR system in blue. A – whole genome representation, B – fragment of genomes near MgaS6I genomic context.

References

1. Darling AE, Mau B, Perna NT (2010) progressiveMauve: Multiple Genome Alignment with Gene Gain, Loss and Rearrangement. PLOS ONE 5(6): e11147. https://doi.org/10.1371/journal.pone.0011147
