# Supplementary material for: Role of DNA modifications in Mycoplasma gallisepticum
Source: PLoS One. 2022 Nov 22;17(11):e0277819. doi: 10.1371/journal.pone.0277819 (PMC9681074; doi:10.1371/journal.pone.0277819)
Supplement: Attachment — Submitted filename: Response to Reviewers.docx [file pone.0277819.s003.docx]

Dear Editor and Reviewer!

Please accept the revised manuscript «Role of DNA modifications in *Mycoplasma gallisepticum*» of the following authors: Tatiana Semashko, Alexander Arzamasov, Daria Evsyutina, Irina Garanina, Daria Matyushkina, Valentina Ladygina, Olga Pobeguts, Gleb Fisunov and Vadim Govorun for consideration as a publication in PLOS ONE journal.

Thank you for your time and for your appreciation of our work. We have corrected our manuscript in accordance with all your comments and suggestions. We hope you find our manuscript suitable for publication.

**Response to Editor.**

1. Please address comments made by the reviewer and myself. Most comments ask for clarification of statements made in the manuscript.

2. Reviewer 1 requests additional informatics work to address the probability of finding a methylation motif in a promoter region normally, given the GC content of the organism. Because this information is important to support arguments made in the manuscript, this analysis should be performed and the results included in your revised manuscript.

We have performed the requested calculations and corrected the text of the manuscript in accordance with results.

3. Include additional evidence to support your suggestion that the RM system was acquired by horizontal gene transfer in your revised manuscript.

We included supplementary figure with comparing genomes of different strains, extended paragraph “RM systems in *M. gallisepticum* and their functionality” in the Results and extended the reasons of our suggestion that the RM system was acquired by horizontal gene transfer in the Discussion.

Additional Editor Comments:

Lines 176-177. The m6A modification and DNA modification motif ANCNNNNCCT were identified only in the WT strain, and the ΔS.MgaS6I strain lacked them. I do not understand why the DNA modification motif ANCNNNNCCT was not identified in the ΔS.MgaS6I strain.

I did not understand the meaning of the statement in lines 177-178, "This indicates that the RM system common for M. gallisepticum strains is nonfunctional, unless in the S6 strain." Are you suggesting that the common RM system is functional in the presence of the unique RM system in MgaS6I?

Thank you for your comments. Both these statements are the results of mistakes. We fixed them.

**Response to Reviewer.**

Major comments

1. It is my understanding that SMRT sequencing only is able to detect m6A modifications on native DNA, and special procedures have to be performed to identify m5C methylations. The authors should comment on whether they performed these procedures, otherwise we don’t know if they missed m5C modifications or not.

Yes, you are right. For identification of DNA modification using SMRT sequencing without additional special procedures, 25x sequencing fold coverage is required for the identification of 4-methylcytosine, glucosylated 5-hydroxymethylcytosine, 6-methyladenine and 8-oxoguanine; 250x sequencing fold coverage is required for the identification of 5-methylcytosine and 5-hydroxymethylcytosine [https://www.pacb.com/wp-content/uploads/2015/09/WP_Detecting_DNA_Base_Modifications_Using_SMRT_Sequencing.pdf]. In our study the coverage of the genome per strand was approximately 90× for WT *M. gallisepticum* S6 and approximately 110× for the ΔS.MgaS6I strain. We didn’t find annotated m5C-methyltransferases in *M. gallisepticum* genome and didn’t identify m5C modifications. But if m5C-methyltransferases were present, we would have seen a not very significant signal for m5C modifications in our experiment.

2. One of the pieces of data the authors present is the general lack of methylation sites within promoter regions. However, it’s not clear if that has occurred randomly or it has been selected for. I would have liked to have seen some informatic work to give an indication on what the probability of finding a methylation motif in a promoter region would be normally, given the GC content of the organism. Is the actual occurrence less than expected by random chance? Or is it that the genome has reduced so much and the motif itself is not simple enough where just randomly the motif doesn’t occur in those spots. Basically, I want to know if nature has selected for removal of methylation motifs from promoters because it could be disruptive. Without this analysis the authors cannot state that methylation sites are “underrepresented” in these places as they do later in the manuscript. They could only say they were not detected. We need to know what the actual expected representation would be to know if the sites are actually underrepresented.

We thank the reviewer for pointing this out. In previous version of manuscript we used two-tailed Fisher’s exact test to validate the observed number of ANCNNNNCCT motif in promoter’s regions compared to occurrence it across whole genome. The promoter sequences in M. gallisepticum S6 were determined in our previous work [Fisunov GY et. al. Reconstruction of Transcription Control Networks in Mollicutes by High-Throughput Identification of Promoters. Front Microbiol. 2016 Dec 6;7:1977]. Now according your suggestion we tried to estimate motif underrepresentation in the promoter regions through ratio of observed over expected motif counts. There are several methods to estimate the expected frequency of short oligonucleotides in the genome. The simplest ones are based on the frequencies of mono- or dinucleotides in the genome. More complex methods use the maximal order Markov model or method by Samuel Karlin [Karlin S, Cardon LR. Computational DNA sequence analysis. Annu Rev Microbiol. 1994;48:619-54.], [Elhai J. Determination of bias in the relative abundance of oligonucleotides in DNA sequences. J Comput Biol. 2001;8(2):151-75.]. We calculated ratio of observed over expected motif through frequencies of each nucleotide in *M. gallisepticum* S6 genome and promoter regions. For whole genome, ratio was 1.29; for promoter regions - 0.2. It means there is no ANCNNNNCCT avoidance in whole genome of M. gallisepticum S6, but it probably exists in promoter regions. We also estimated enrichment ratio of ANCNNNNCCT in set of promoter sequences and in 100 sets of random generated sequences with the same length and GC content of the M. gallisepticum S6. In this case enrichment ratio was 0.49±0.11.

3. As a side note, I appreciate that the authors did not try to over-extend their transcriptional change data. It would have been easy to present some of those transcriptional changes as significant in the figure, but they relied on the statistics and showed that most genes showed no significant change in gene expression. That’s good science.

Thank you!

4. Line 320 – the authors state the RM system was acquired by horizontal gene transfer. That’s a big statement to make. I would make sure to provide all the evidence that supports it. The only thing that comes to mind right now is that it’s present in the S6 strain and none of the others. But there are things that could be done to back up that statement.

The MgaS6I system does not have close homologs in other *M. gallisepticum* strains. Additionally, it has a unique location in a genomic context compared to other strains. We added figure S1 with alignments of genomes of *M. gallisepticum* different strains. We extended of arguments for our suggestion that the RM system was acquired by horizontal gene transfer in text of the manuscript: in the paragraph “RM systems in *M. gallisepticum* and their functionality” in the Results and in the second paragraph of the Discussion.

5. Line 328 – honestly, I just don’t understand what the authors are talking about in this paragraph. Please clarify.

In this paragraph we discuss underrepresentation of recognition sites in whole genome of *M. gallisepticum* S6. Sites are not underrepresented comparing with genomes of other strains. We have rewritten this paragraph.

Minor comments:

Line 16-17 – poor grammar, should be “investigated DNA modifications of the model”

Thank you, we fixed it.

Line 178-179 – I’m confused by this sentence. The authors state that the RM system common to the different strains of M. gallisepticum is non-functional, which makes sense based on the mutations in this system, but then include the clause “unless in the S6 strain”. It’s this last part I don’t understand. I thought the point was that the unique system is responsible for the methylation, but that clause indicates the common system is responsible for the methylation.

Thank you, we fixed this typo.

Line 195 – I believe there is a typo, and it should be MgaS6I instead of MhaS6I

Thank you, we fixed it.

Best regards,

Tatiana Semashko

Address: Nauchnyj proezd 18, Moscow, Russia

Email: t.semashko@gmail.com
